# Clinical utility of CLL-IPI scoring system in Pakistani Chronic Lymphocytic Patients: A single center experience

**DOI:** 10.12669/pjms.40.4.8703

**Published:** 2024

**Authors:** Aisha Hameed, Nadia Sajid, Muhammad Fayyaz, Shagufta Khaliq

**Affiliations:** 1 Aisha Hameed, MBBS, M.Phil. Department of Hematology, University of Health Sciences, Lahore, Punjab, Pakistan. Department of Pathology, Gujranwala Medical College/Teaching Hospital, Gujranwala, Punjab, Pakistan; 2 Nadia Sajid, MBBS, FCPS. Department of Clinical Hematology, Institute of Nuclear Medicine and Oncology (INMOL) Cancer Hospital, Lahore, Punjab, Pakistan; 3 Muhammad Fayyaz, MS. Department of Pathology, Gujranwala Medical College/Teaching Hospital, Gujranwala, Punjab, Pakistan; 4 Shagufta Khaliq, M.Phil, PhD. Department of Human Genetics and Molecular Biology, University of Health Sciences, Lahore, Punjab, Pakistan. The University of Lahore, Lahore, Pakistan

**Keywords:** CLL, CLL-IPI, Overall survival, Pakistan

## Abstract

**Objectives:**

To determine validity of the CLL International prognostic index (IPI) scoring system in Pakistani chronic lymphocytic leukemia patients, as the validity and universal applicability of various prognostic scoring systems such as the CLL-IPI remains a challenge, particularly in under-developed countries like Pakistan.

**Methods:**

This prospective single center study was conducted at Department of Hematology, University of Health Sciences, Lahore and included sixty patients with CLL diagnosed between July, 2019 to July, 2022. Patients were followed for a period of two years and 02 year overall survival (OS) was noted. Risk stratification was conducted according to CLL-IPI prognostic model.

**Results:**

Among 60 patients, the mean age was 60±11years. Advanced Binet stage B+C and elevated β2-microglobulin >3.5mg/L was observed in 73.3% and 38.3% patients respectively. The estimated median 02 years OS was 16.5 months (95% CI: 10-20 months). In total, 40 of 60 CLL patients (67%) were accessible for follow-up analyses. For the present CLL cohort, 25% patients (n = 10) were classified as CLL-IPI low risk and intermediate risk group, 35% (n = 14) as high risk and 15% (n = 06) as very high-risk group. However, this classification of patients according to CLL-IPI did not yield significant differences in terms of OS (*p* = 0.24), although the median OS of CLL-IPI very high-risk group was noted as only six months and was not reached for low and intermediate risk groups.

**Conclusion:**

To conclude, clinical validation of CLL-IPI scoring system could not be established for the present CLL cohort and needs to be evaluated in further studies with larger sample size.

## INTRODUCTION

Chronic lymphocytic leukemia (CLL) is characterized by clonal expansion of small relatively monomorphic B lymphocytes in peripheral blood, bone marrow and other secondary lymphoid organs.^1^ CLL represents a rare hematological malignancy in Asians (<5% of all leukemias) whereas it is 10-fold more common in the Western hemisphere, accounting for >30% of all leukemia cases.^2^,^3^ The clinical course of CLL is very heterogeneous in nature as reflected by two extremes where some patients show indolent disease and survive for decades with minimal or no therapeutic intervention, while others may experience rapid disease progression even after aggressive treatment.^4^

The Rai and Binet staging systems which were published about forty years ago, are still considered gold standard for stratification of CLL patients in routine practice, especially in Asian countries.^4^,^5^ These systems are dependent on clinical features and simple laboratory test, so easy to apply in resource limited set ups. But these conventional staging systems are not able to accurately predict disease progression especially for early stages and also do not include *TP53* aberrations, immunoglobulin heavy chain variable region (*IGHV*) gene mutation status and other indicators that have clear prognostic significance for CLL.^6^ To overcome these limitations, extensive research has been conducted globally and new prognostic models including MD Anderson Cancer Center (MDACC), German CLL Study Group (GCLLSG), and CLL International Prognostic Index (CLL-IPI) have been evaluated in various CLL cohorts in Western countries.^7^

The most validated scoring approach in clinical management, the CLL-IPI, incorporates cytogenetic and clinical data into a prognostic model. It has five independent variables, including *TP53* and *IGHV* genes mutational status, age, clinical stage and β2M concentration, classifying patients into four risk categories (low, intermediate, high and very-high risk).^8^ For patients to be in very-high risk category, *TP53* aberration should be present, highlighting importance of *TP53* status in CLL. In fact, IWCLL guidelines also recommend for evaluation of *TP53* aberrations and *IGHV* mutational status at diagnosis.^1^,^7^ Use of CLL-IPI in predicting treatment free survival (TFS) and overall survival (OS) has been validated in meta-analysis based clinical trials and few real world CLL sample sets of mainly Caucasian and East Asian in origin.^8^-^10^

However, before CLL-IPI can be regarded as a standard and universal prognostication system for CLL, its reproducibility and clinical utility should be evaluated in CLL patient cohorts of different ethnic origins including the Indo-Pak region. Considering the ethnic differences and population-based variability and the populations on which these prognostic scores have been developed, it has become essential to validate the prognostic significance of CLL-IPI in Pakistani patients with CLL. Therefore, the present study was designed to evaluate the applicability and validity of this score in Pakistani CLL patients.

## METHODS

This prospective study included 60 patients diagnosed with typical CLL presenting at a single cancer specialty center (INMOL Cancer Hospital) in Lahore, Punjab, Pakistan between July, 2019 to July, 2022. All patients had newly diagnosed CLL according to the IWCLL criteria, based on persistent lymphocytosis, typical lymphocyte morphology on peripheral blood smears, and immunophenotyping results.^1^ Baseline clinical and laboratory characteristics were obtained from the patients’ charts. The clinical stage of the patients was determined according to the Binet staging system.^5^ Patients were followed from time of diagnosis to death or last follow up (up to a maximum of two years), whichever occurred first, termed two years overall survival (OS). Peripheral blood samples were collected at diagnosis before initiation of treatment. LDH and β2-microglobulin levels were measured by ELISA using commercially available kit (Randox and R&D Systems, USA, respectively) as these are the most common conventional serum parameters used as prognostic biomarkers. Analysis of *IGHV* and *TP53* genes was performed in accordance with the protocols described previously.^11^,^12^

### Ethical Approval

All patients received a detailed explanation of the study and provided written informed consent for the data/sample collection and testing, in accordance with the Declaration of Helsinki guidelines and under the study protocol approved by the Advanced studies & Research Board (ASRB) (No: UHS/Education/126-20/568, dated 20-02-2020) of the University of the Health Sciences (Lahore, Pakistan).

### Statistical Analysis

Patient characteristics were summarized using descriptive statistics in the form of frequencies and percentages (categorical variables) or in the form of mean ± standard deviation (normally distributed data) or median and range (skewed data) for continuous variables. Prognostic significance of different variables was determined by using Cox proportional hazard models. Survival curves were plotted using Kaplan-Meier estimates and log-rank statistics were used to evaluate differences between different CLL-IPI risk categories. All *p-*values were two-sided and considered statistically significant when <0.05. Statistical analyses were performed using Statistical Package for Social Sciences (SPSS) version 20 for windows and GraphPad Prism 8.0.

## RESULTS

In this series of 60 CLL patients, the mean age at presentation was 60 years with 18.3% patients being >65 years of age. In terms of sex distribution, a male predominance was observed among CLL patients [males: 48 (80%), and male to female ratio of 4:1]. Clinically, about 2/3 of the patients had advanced stage disease (Binet stage B+C: 44, 73.3%). Supplementary Table-I contains detailed laboratory and clinical features.

**Supplementary Table-I:** Baseline clinical and laboratory features of CLL patients.

Characteristics	CLL patients (n = 60)
Mean age (±SD)	60 (±11) years
Age ≤ 65 years	49 (81.7%)
Age > 65 years	11 (18.3%)
Female	12 (20%)
Male	48 (80%)
B symptoms, n (%)	43 (71.6%)
Lymphadenopathy, n (%)	33 (55%)
Organomegaly, n (%)	27 (45%)
***Binet stage, n (%)***	
A	16 (26.6%)
B	12 (20%)
C	32 (53.3%)
Lymphocytosis ≥30 ×10^9^/L	45 (75%)
Mean Hb (±SD)	10.92 (±2.63) g/dl
Anemia (Hb ≤10g/dL)	26 (43.3%)
Mean platelet count (±SD)	148.84 (±73.42) ×10^9^/L
Thrombocytopenia (Platelets ≤100 ×10^9^/L)	16 (26.6%)
Median β2 microglobulin (IQR)	3.16 (2.20) mg/L
Elevated β2 microglobulin ≥3.5 mg/L	23 (38.3%)
Median LDH (IQR)	334 (221.25) U/L
***IGHV mutational status, n = 40 (%)***	
Mutated	23 (57.5%)
Unmutated	17 (42.5%)
***TP53 mutations, n=46 (%)***	
Detected	08 (17.4%)
Del 17p, n=17 (%)	
Detected	02 (11.8%)

CLL, Chronic lymphocytic leukemia; del 17p, deletion 17p; Hb, Hemoglobin; IGHV, Immunoglobulin heavy variable gene; IQR, Inter quartile range; LDH, Lactate dehydrogenase; TP53, Tumor protein P53.

In total, 40 of 60 CLL patients (67%) were accessible for follow-up analyses, of whom 12 (30%) died due to CLL related (disease progression and chemotherapy mediated complications) or unrelated (comorbidities) causes during the period of observation. The estimated median two years OS was 16.5 months (95% CI: 10-20 months).

Prognostic impact of the variables included in CLL-IPI model e.g., age > 65 years, Binet stage B & C, β2-microglobulin and genetic factors was evaluated using Cox proportional hazard models. By univariate analysis, significant or near significant risk factors associated with poor survival were elevated β2-microglobulin levels (HR = 3.49, 95% CI: 1.05-11.60), and mutated *TP53* (HR = 3.20, 95% CI: 0.95-10.73) (Table-I).

**Table-I:** Cox univariate analysis of 02-years OS in CLL patients (n=40).

Study variables	HR	95% CI	p-value^a^
***Cox univariate analysis***			
***Age***			
<65 years (n = 33)	1.00	-	0.23
>65 years (n = 07)	2.08	0.62-6.94
***Binet stage***			
A (n = 10)		-	
B+C (n = 30)	4.26	0.54-33.09	0.16
***β2 microglobulin***			
<3.5 mg/L (n = 25)	1.00	-	0.04
≥3.5 mg/L (n = 15)	3.49	1.05-11.60
***IGHV mutation status***			
M-CLL (n = 19)	1.00	-	0.11
UM-CLL (n = 17)	2.71	0.81-9.03
***TP53 status***			
Wild-type TP53 (n = 33)	1.00	-	0.06
Mutated TP53 (n = 07)	3.20	0.95-10.73	

95% CI, 95% confidence interval; CLL, Chronic lymphocytic leukemia; HR, hazard ratio; IGHV, Immunoglobulin heavy variable gene; M, mutated; TP53, Tumor protein P53, UM, Un-mutated. ap-value, probability that the hazard ratio = 01 (null hypothesis). p-value in bold indicates statistical significance.

**Fig.1:**
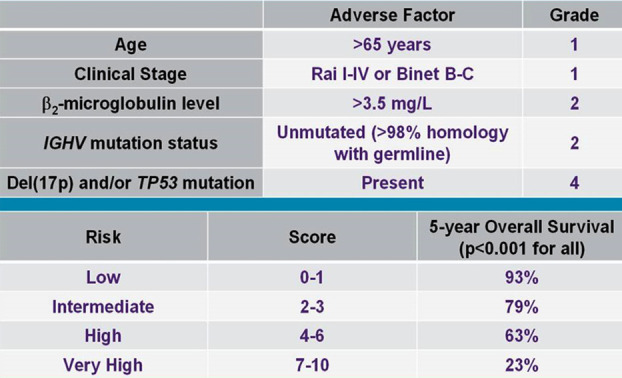
Grading and five years overall survival according to CLL-IPI.^8^

In multivariate analysis of the risk variables with a *p* < 0.1 in univariate analysis, no factor persisted as significant independent predictor of poor OS in our patients (Table-II).The prognostic ability of CLL-IPI was measured in terms of OS and the scores were calculated as per the weighted grades assigned to each variable involved in score categorization (Fig.1).

**Table-II:** Cox multivariate analysis of 02-years OS in CLL patients.

Study variables	HR	95% CI	p-value^a^
***β2 microglobulin***			
<3.5 mg/L (n = 25)	1.00	-	0.08
≥3.5 mg/L (n = 15)	2.95	0.85-10.20
***TP53 status***			
Wild-type TP53 (n = 33)	1.00	-	0.17
Mutated TP53 (n = 07)	2.35	0.67-8.24	

95% CI, 95% confidence interval; HR, hazard ratio; IGHV, Immunoglobulin heavy variable gene; M, mutated; TP53, Tumor protein P53, UM, Un-mutated. ap-value, probability that the hazard ratio = 01 (null hypothesis). p-value in bold indicates statistical significance.

**Fig.2:**
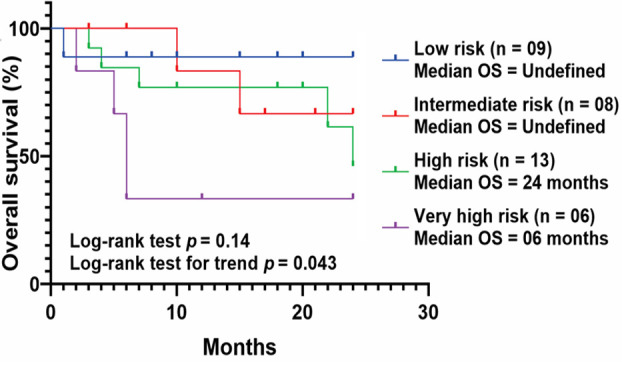
Kaplan-Meier estimates for two years overall survival (OS) in CLL patients classified into different CLL-IPI index groups.

For the present CLL cohort, 25% patients (n = 10) were classified as CLL-IPI low risk group, another 25% (n = 10) as intermediate risk, 35% (n = 14) as high risk and 15% (n = 06) as very high-risk group. However, this classification of patients according to CLL-IPI did not yield significant differences in terms of OS (*p* = 0.24), although the median OS of CLL-IPI very high-risk group was noted as only six months and with the increasing risk category from low to very high risk, a significantly decreasing trend in OS was observed (Fig.2).

## DISCUSSION

As the literature has definitely confirmed about heterogeneity of CLL, so there is a dire need for predictive and prognostic biomarkers to guide patient risk stratification and treatment initiation accordingly.^13^ In addition, conventional staging systems Rai and Binet have become of limited use in patient risk stratification and new prognostic scoring system is needed to stratify the risk of CLL clinically.^6^

Many prognostic scoring systems have been published by different CLL working groups from various global ethnicities, the most renowned of which being CLL-IPI, which was established by the international consortium on CLL.^8^ Moreover, CLL displays marked differences in incidence rates, demographic, clinicopathological and genetic features in persons from different ethnic groups. This ethnic and geographic diversity is especially more pronounced in comparisons of Asian CLL (including predominantly Pakistani and Indian patients) and European CLL (discussed later).^2^,^6^ As scoring systems including CLL-IPI have been developed and evaluated on western populations, so there is need to validate it in geographical novel sample set of Pakistani CLL patients.

In the present study, prognostic value of the CLL-IPI model was evaluated but surprisingly risk categorization of study patients according to CLL-IPI scoring system did not prove statistically significant in the prediction of OS.

In this study, the mean age at diagnosis was 60 years with only 18.3% patients being > 65 years of age. This age distribution in CLL is consistent with that reported in other Pakistani (50.9-60 years)^14^,^15^ and Indian as well as Chinese (60 years) studies.^3^,^6^,^16^,^17^ In comparison, median age is reached almost a decade later in Caucasians (69-70 years).^18^,^19^ But age > 65 years in present study, was not significantly associated with dismal survival that is in line with Indian CLL-IPI validation study but differs from Chinese CLL-IPI study.^6^,^16^

In our study, about 2/3 (73.3%) of the CLL patients presented with advanced Binet stages B and C that seems to be a hallmark of this disease from under-developed regions, as confirmed by other Indo-Pakistani studies.^14^,^17^ While, representation of advanced Binet stages among CLL patients decreases sharply for Americans (24.7%) 19 and especially for Europeans (18.8%).^20^ However, an unexpected finding was no significant impact of Binet staging on survival outcomes in our study, which is in line with a local study by Mahmood et al but in contrast to Indian studies.^16^,^17^,^21^ Of the five parameters involved in categorization of CLL-IPI score, only β2-microglobulin was significantly associated with poor two years OS in univariate analysis as already suggested, although the relative percentage of CLL patients with elevated β2-microglobulin ranges from low (10.7%) in Europeans,^20^ moderate (37.7-38.3%) in Pakistanis^21^ and high (70-75.4%) in Indians.^16^,^17^

This study is the first ever study to evaluate clinical validity of CLL-IPI scoring system in any Pakistani CLL sample set. Our findings show that CLL-IPI system-based risk groups of patients did not have significantly different overall survival curves. On the contrary, in an Indian validation study, CLL-IPI risk groups reflected significantly different OS periods in a relatively larger sample set of CLL.^16^ Similarly, CLL-IPI proved to be superior in predicting OS and time to first treatment (TTFT) in a Spanish cohort.^22^ A Chinese and recent Canadian study also demonstrated the validity of the CLL-IPI score in correlation to survival.^6^,^10^ The differences in the results obtained in our series could be due to lower number of patients in our study as compared to other studies performed, and also due to short follow-up, there are not enough events in our cohort.

### Limitations

It includes small sample size with short follow up period which is due to rarity of CLL in our country (5% of hematological malignancies), Covid-19 pandemic during study period and time-constraints. Second, it is a single center study and its results may not be generalized to all CLL patients.

## CONCLUSIONS

In conclusion, despite its inherent limits, this is the first analysis for the validation of CLL-IPI prognostic model in any Pakistani CLL cohort. However, the prognostic significance and subsequently, clinical utility of CLL-IPI could not be demonstrated in terms of survival outcomes and needs to be elucidated in further studies with larger sample size that might have resulted in finer determination of distinct survival patterns for CLL-IPI risk groups in this study.

### Recommendations

Multicenter studies are required to determine whether CLL-IPI model is applicable and feasible in our CLL patients. Moreover, considering the diverse clinicopathological features of our CLL patients in comparison to Western CLL, existing scoring systems can be modified accordingly.

### Authors Contributions:

**AH:** Entire research work (conception/design, data collection/ analysis and interpretation),

**MF:** Methodology.

**NS:** Critical Review/editing and supervision.

**SK:** Final approval.

**AH** & **SK:** Responsible for integrity of the work.

All authors contributed to the study conception, design, read and approved the final manuscript.
